# 7-Amino-1,8-naphthyridin-2(1*H*)-one monohydrate

**DOI:** 10.1107/S1600536811033599

**Published:** 2011-08-31

**Authors:** Zhen Li

**Affiliations:** a Faculty of Chemistry and Chemical Engineering, Yunnan Normal University, Kunming 650092, People’s Republic of China

## Abstract

In the crystal structure of the title compound, C_8_H_7_N_3_O·H_2_O, adjacent organic mol­ecules are linked together into a tape along the *a* axis through N—H⋯N and N—H⋯O hydrogen bonds. On the other hand, water mol­ecules are linked together to form a chain along the *b* axis through O—H⋯O hydrogen bonds. The water chains and the organic mol­ecular tapes are further connected by inter­molecular O—H⋯O hydrogen bonds, forming a three-dimensional network. In addition, a π–π stacking inter­action between the 1,8-naphthyridine ring systems with an inter­planar separation of 3.246 (1) Å and a centroid–centroid distance of 3.825 (2) Å is observed.

## Related literature

For applications of 1,8-naphthyridine and its derivatives in coordination chemistry, see: Oskui *et al.* (1999 ▶); Nakatani *et al.* (2003 ▶); Fang *et al.* (2004 ▶); Sinha *et al.* (2009 ▶); Fu *et al.* (2009 ▶, 2010 ▶). For related structures of 1,8-naphthyridine derivatives, see: Goswami *et al.* (2007 ▶). For the synthesis of the title compound, see: Newcome *et al.* (1981 ▶).
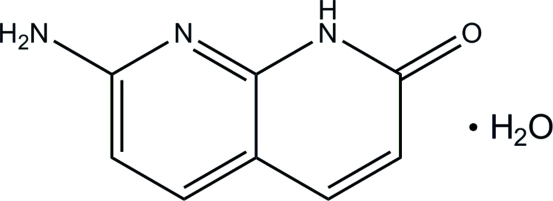


## Experimental

### 

#### Crystal data

C_8_H_7_N_3_O·H_2_O
*M* *_r_* = 179.18
Monoclinic, 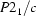

*a* = 9.5413 (9) Å
*b* = 17.1560 (16) Å
*c* = 4.9954 (4) Å
β = 95.19 (2)°
*V* = 814.34 (13) Å^3^
*Z* = 4
Mo *K*α radiation
μ = 0.11 mm^−1^
*T* = 113 K
0.22 × 0.20 × 0.02 mm

#### Data collection

Bruker APEX CCD diffractometer
Absorption correction: multi-scan (*SADABS*; Sheldrick, 1996 ▶) *T*_min_ = 0.977, *T*_max_ = 0.998
6502 measured reflections
1432 independent reflections
906 reflections with *I* > 2σ(*I*)
*R*_int_ = 0.085

#### Refinement

*R*[*F* ^2^ > 2σ(*F* ^2^)] = 0.063
*wR*(*F* ^2^) = 0.171
*S* = 1.00
1432 reflections
118 parameters
H-atom parameters constrained
Δρ_max_ = 0.30 e Å^−3^
Δρ_min_ = −0.28 e Å^−3^

### 

Data collection: *SMART* (Bruker, 2004 ▶); cell refinement: *SAINT* (Bruker, 2004 ▶); data reduction: *SAINT*; program(s) used to solve structure: *SHELXS97* (Sheldrick, 2008 ▶); program(s) used to refine structure: *SHELXL97* (Sheldrick, 2008 ▶); molecular graphics: *DIAMOND* (Brandenburg, 1999 ▶); software used to prepare material for publication: *SHELXTL* (Sheldrick, 2008 ▶).

## Supplementary Material

Crystal structure: contains datablock(s) I, global. DOI: 10.1107/S1600536811033599/is2766sup1.cif

Structure factors: contains datablock(s) I. DOI: 10.1107/S1600536811033599/is2766Isup2.hkl

Supplementary material file. DOI: 10.1107/S1600536811033599/is2766Isup3.cdx

Supplementary material file. DOI: 10.1107/S1600536811033599/is2766Isup4.cml

Additional supplementary materials:  crystallographic information; 3D view; checkCIF report

## Figures and Tables

**Table 1:** Hydrogen-bond geometry (Å, °)

*D*—H⋯*A*	*D*—H	H⋯*A*	*D*⋯*A*	*D*—H⋯*A*
N1—H1*A*⋯O1^i^	0.86	2.00	2.853 (3)	175
N1—H1*B*⋯O1^ii^	0.86	2.28	2.989 (3)	140
N3—H1⋯N2^i^	0.86	2.18	3.040 (3)	178
O2—H2*A*⋯O2^iii^	0.85	1.93	2.7758 (18)	179
O2—H2*B*⋯O1	0.85	1.97	2.823 (3)	178

Symmetry codes: (i) 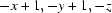; (ii) 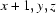; (iii) 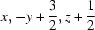.

**Fig. 1.**
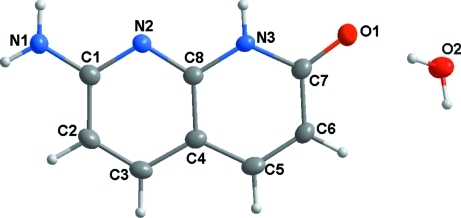
The asymmetric unit of the title compound, with displacement ellipsoids drawn at the 30% probability level.

**Fig. 2.**
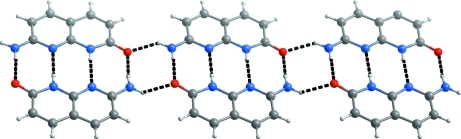
The tape structure of the organic molecules propagating in the a axis direction through N—H···N and N—H···O hydrogen bonds.

**Fig. 3.**
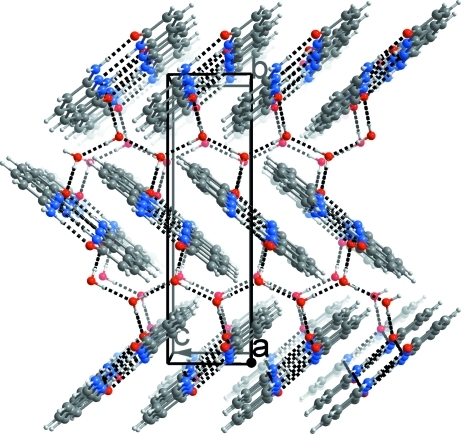
The three-dimensional packing of the title compound viewed along the a axis, showing the hydrogen bonds with dashed lines.

## supplementary crystallographic information

### Comment

The 1,8-naphthyridine and its derivatives have attracted considerable attention as an polydentate nitrogen-donor ligand, since these ligands are linked to metals with several coordination modes such as monodentate and chelating bidentate fashion with short metal-metal bonds (Oskui *et al.*, 1999). In addition, the metal 1,8-naphthyridine complexes can exhibit biocompatibility, interesting catalytic and fluorescence properties (Nakatani *et al.*, 2003; Fang *et al.*, 2004; Sinha *et al.*, 2009). Previously, we have concentrated our investigation on the chemistry of 1,8-naphthyridine derivatives and related metal coordination compounds (Fu *et al.*, 2009, 2010). Herein, we report herein the crystal structure of the title compound. Although the synthesis of the compound 7-amino-1,8-naphthyridin-2(1*H*)-one has been reported (Newcome *et al.*, 1981), no crystallographic study has been reported on this ligand until now.

The asymmetric unit of the title compound contains a crystallographically independent molecules and one water molecule (Fig. 1), in which the bond lengths and angles are generally within normal ranges in accordance with the corresponding values reported (Goswami *et al.*, 2007). The organic molecule is nearly planar with an r.m.s. deviation of 0.0146 Å. In the crystal structure, adjacent organic molecules are joined together into a one-dimensional tape propagating in the *a* axis direction (Fig. 2) through N—H···N and N—H···O hydrogen bonds (Table 1). Neighboring water molecules joined together to form a one-dimensional water chain extending along the *b* axis (Fig. 3) through OW—H···OW hydrogen bonds. These one-dimensional water chains are further connected to the adjacent one-dimensional tape motifs, forming a three-dimensional network (Fig. 3) by intermolecular OW—H···O hydrogen bonds (Table 1). In addition, a π–π stacking interaction between 1,8-naphthyridine rings may further stabilize the structure; the interplanar distance and the centrioid-centrioid distance are 3.246 (1) and 3.825 (2) Å, respectively.

### Experimental

The 7-amino-1,8-naphthyridin-2(1*H*)-one was synthesized according to the literature method (Newcome *et al.*, 1981). The colorless crystals of the title compound suitable for X-ray diffraction were obtained by vapor diffusion of diethyl ether into the solution of the product 7-amino-1,8-naphthyridin-2(1*H*)-one in CH_3_CN-MeOH (*v*:*v* = 4:1).

### Refinement

All H atoms were placed in idealized positions (O—H = 0.85 Å, N—H = 0.86 Å and C—H = 0.95 Å) and refined as riding atoms with *U*_iso_(H) = 1.2*U*_eq_(C, N) and *U*_iso_(H) = 1.5*U*_eq_(O).

### Crystal data

**Table:**

C_8_H_7_N_3_O·H_2_O	*F*(000) = 376
*M**_r_* = 179.18	*D*_x_ = 1.461 Mg m^−^^3^
Monoclinic, *P*2_1_/*c*	Mo *K*α radiation, λ = 0.71073 Å
Hall symbol: -P 2ybc	Cell parameters from 2181 reflections
*a* = 9.5413 (9) Å	θ = 2.1–27.8°
*b* = 17.1560 (16) Å	µ = 0.11 mm^−^^1^
*c* = 4.9954 (4) Å	*T* = 113 K
β = 95.19 (2)°	Prism, colorless
*V* = 814.34 (13) Å^3^	0.22 × 0.20 × 0.02 mm
*Z* = 4	

### Data collection

**Table:**

Bruker APEX CCD diffractometer	1432 independent reflections
Radiation source: fine-focus sealed tube	906 reflections with *I* > 2σ(*I*)
graphite	*R*_int_ = 0.085
Detector resolution: 14.63 pixels mm^-1^	θ_max_ = 25.0°, θ_min_ = 2.1°
ω scan	*h* = −11→11
Absorption correction: multi-scan (*SADABS*; Sheldrick, 1996)	*k* = −20→20
*T*_min_ = 0.977, *T*_max_ = 0.998	*l* = −5→5
6502 measured reflections	

### Refinement

**Table:**

Refinement on *F*^2^	Primary atom site location: structure-invariant direct methods
Least-squares matrix: full	Secondary atom site location: difference Fourier map
*R*[*F*^2^ > 2σ(*F*^2^)] = 0.063	Hydrogen site location: inferred from neighbouring sites
*wR*(*F*^2^) = 0.171	H-atom parameters constrained
*S* = 1.00	*w* = 1/[σ^2^(*F*_o_^2^) + (0.0752*P*)^2^] where *P* = (*F*_o_^2^ + 2*F*_c_^2^)/3
1432 reflections	(Δ/σ)_max_ < 0.001
118 parameters	Δρ_max_ = 0.30 e Å^−^^3^
0 restraints	Δρ_min_ = −0.28 e Å^−^^3^

### Special details

**Table:**

Geometry. All e.s.d.'s (except the e.s.d. in the dihedral angle between two l.s. planes) are estimated using the full covariance matrix. The cell e.s.d.'s are taken into account individually in the estimation of e.s.d.'s in distances, angles and torsion angles; correlations between e.s.d.'s in cell parameters are only used when they are defined by crystal symmetry. An approximate (isotropic) treatment of cell e.s.d.'s is used for estimating e.s.d.'s involving l.s. planes.
Refinement. Refinement of *F*^2^ against ALL reflections. The weighted *R*-factor *wR* and goodness of fit *S* are based on *F*^2^, conventional *R*-factors *R* are based on *F*, with *F* set to zero for negative *F*^2^. The threshold expression of *F*^2^ > σ(*F*^2^) is used only for calculating *R*-factors(gt) *etc*. and is not relevant to the choice of reflections for refinement. *R*-factors based on *F*^2^ are statistically about twice as large as those based on *F*, and *R*- factors based on ALL data will be even larger.

### Fractional atomic coordinates and isotropic or equivalent isotropic displacement parameters (Å^2^)

**Table:**

	*x*	*y*	*z*	*U*_iso_*/*U*_eq_	
O1	0.1654 (2)	0.58746 (12)	0.1928 (4)	0.0401 (6)	
O2	−0.0186 (2)	0.71471 (12)	0.0843 (4)	0.0461 (7)	
H2A	−0.0189	0.7369	0.2365	0.055*	
H2B	0.0386	0.6772	0.1179	0.055*	
N1	0.8793 (2)	0.52074 (14)	0.2402 (5)	0.0381 (7)	
H1A	0.8606	0.4889	0.1089	0.046*	
H1B	0.9651	0.5281	0.3036	0.046*	
N2	0.6422 (2)	0.54442 (13)	0.2362 (4)	0.0286 (6)	
N3	0.4034 (2)	0.56892 (13)	0.2314 (4)	0.0290 (6)	
H1	0.3930	0.5370	0.0984	0.035*	
C1	0.7755 (3)	0.55862 (17)	0.3454 (6)	0.0333 (7)	
C2	0.8036 (3)	0.61017 (18)	0.5674 (6)	0.0364 (8)	
H2	0.8978	0.6187	0.6399	0.044*	
C3	0.6969 (3)	0.64711 (17)	0.6756 (6)	0.0348 (8)	
H3	0.7160	0.6817	0.8229	0.042*	
C4	0.5572 (3)	0.63391 (17)	0.5685 (6)	0.0322 (7)	
C5	0.4358 (3)	0.66882 (16)	0.6616 (6)	0.0363 (8)	
H5	0.4467	0.7030	0.8116	0.044*	
C6	0.3041 (3)	0.65478 (17)	0.5423 (6)	0.0344 (8)	
H6	0.2250	0.6792	0.6093	0.041*	
C7	0.2846 (3)	0.60331 (16)	0.3161 (6)	0.0335 (8)	
C8	0.5372 (3)	0.58251 (15)	0.3459 (6)	0.0285 (7)	

### Atomic displacement parameters (Å^2^)

**Table:**

	*U*^11^	*U*^22^	*U*^33^	*U*^12^	*U*^13^	*U*^23^
O1	0.0361 (12)	0.0370 (14)	0.0470 (14)	0.0041 (9)	0.0037 (10)	0.0006 (10)
O2	0.0615 (15)	0.0333 (13)	0.0421 (14)	0.0105 (11)	−0.0025 (12)	0.0020 (10)
N1	0.0301 (13)	0.0420 (17)	0.0423 (18)	−0.0001 (11)	0.0036 (12)	−0.0050 (13)
N2	0.0308 (13)	0.0253 (13)	0.0296 (15)	−0.0033 (10)	0.0024 (11)	0.0030 (11)
N3	0.0345 (13)	0.0261 (13)	0.0265 (15)	0.0028 (10)	0.0032 (11)	−0.0016 (10)
C1	0.0382 (17)	0.0269 (17)	0.0348 (19)	−0.0047 (14)	0.0025 (14)	0.0073 (14)
C2	0.0387 (17)	0.0306 (18)	0.039 (2)	−0.0079 (13)	−0.0035 (15)	0.0043 (14)
C3	0.0460 (18)	0.0258 (17)	0.0317 (19)	−0.0060 (14)	−0.0013 (15)	0.0006 (13)
C4	0.0431 (17)	0.0230 (16)	0.0305 (19)	0.0002 (13)	0.0042 (14)	0.0037 (13)
C5	0.053 (2)	0.0227 (16)	0.0334 (19)	0.0011 (14)	0.0050 (15)	−0.0003 (14)
C6	0.0389 (17)	0.0268 (17)	0.0385 (19)	0.0054 (13)	0.0099 (15)	0.0023 (14)
C7	0.0392 (17)	0.0243 (16)	0.0377 (19)	0.0039 (13)	0.0066 (15)	0.0055 (14)
C8	0.0364 (16)	0.0192 (15)	0.0300 (18)	−0.0014 (12)	0.0037 (14)	0.0056 (13)

### Geometric parameters (Å, °)

**Table:**

O1—C7	1.273 (3)	C1—C2	1.425 (4)
O2—H2A	0.8500	C2—C3	1.353 (4)
O2—H2B	0.8500	C2—H2	0.9500
N1—C1	1.332 (4)	C3—C4	1.409 (4)
N1—H1A	0.8600	C3—H3	0.9500
N1—H1B	0.8600	C4—C8	1.418 (4)
N2—C8	1.353 (3)	C4—C5	1.419 (4)
N2—C1	1.360 (3)	C5—C6	1.363 (4)
N3—C8	1.370 (3)	C5—H5	0.9500
N3—C7	1.378 (3)	C6—C7	1.433 (4)
N3—H1	0.8600	C6—H6	0.9500
			
H2A—O2—H2B	102.5	C4—C3—H3	120.2
C1—N1—H1A	120.0	C3—C4—C8	116.9 (3)
C1—N1—H1B	120.0	C3—C4—C5	125.4 (3)
H1A—N1—H1B	120.0	C8—C4—C5	117.6 (3)
C8—N2—C1	116.8 (3)	C6—C5—C4	122.0 (3)
C8—N3—C7	124.0 (3)	C6—C5—H5	119.0
C8—N3—H1	118.0	C4—C5—H5	119.0
C7—N3—H1	118.0	C5—C6—C7	120.2 (3)
N1—C1—N2	117.2 (3)	C5—C6—H6	119.9
N1—C1—C2	121.1 (3)	C7—C6—H6	119.9
N2—C1—C2	121.7 (3)	O1—C7—N3	118.9 (3)
C3—C2—C1	120.5 (3)	O1—C7—C6	124.0 (3)
C3—C2—H2	119.8	N3—C7—C6	117.1 (3)
C1—C2—H2	119.8	N2—C8—N3	116.4 (3)
C2—C3—C4	119.5 (3)	N2—C8—C4	124.5 (3)
C2—C3—H3	120.2	N3—C8—C4	119.2 (3)
			
C8—N2—C1—N1	179.3 (3)	C8—N3—C7—C6	−1.6 (4)
C8—N2—C1—C2	1.0 (4)	C5—C6—C7—O1	−179.6 (3)
N1—C1—C2—C3	−178.7 (3)	C5—C6—C7—N3	0.9 (4)
N2—C1—C2—C3	−0.4 (4)	C1—N2—C8—N3	178.8 (2)
C1—C2—C3—C4	0.4 (5)	C1—N2—C8—C4	−1.7 (4)
C2—C3—C4—C8	−1.0 (4)	C7—N3—C8—N2	−179.4 (2)
C2—C3—C4—C5	−179.8 (3)	C7—N3—C8—C4	1.1 (4)
C3—C4—C5—C6	178.1 (3)	C3—C4—C8—N2	1.7 (4)
C8—C4—C5—C6	−0.7 (4)	C5—C4—C8—N2	−179.4 (3)
C4—C5—C6—C7	0.1 (4)	C3—C4—C8—N3	−178.8 (2)
C8—N3—C7—O1	179.0 (2)	C5—C4—C8—N3	0.1 (4)

### Hydrogen-bond geometry (Å, °)

**Table:**

*D*—H···*A*	*D*—H	H···*A*	*D*···*A*	*D*—H···*A*
N1—H1A···O1^i^	0.86	2.00	2.853 (3)	175.
N1—H1B···O1^ii^	0.86	2.28	2.989 (3)	140.
N3—H1···N2^i^	0.86	2.18	3.040 (3)	178.
O2—H2A···O2^iii^	0.85	1.93	2.7758 (18)	179.
O2—H2B···O1	0.85	1.97	2.823 (3)	178.

Symmetry codes: (i) −*x*+1, −*y*+1, −*z*; (ii) *x*+1, *y*, *z*; (iii) *x*, −*y*+3/2, *z*+1/2.

## References

[bb1] Brandenburg, K. (1999). *DIAMOND* Crystal Impact GbR, Bonn, Germany.

[bb2] Bruker (2004). *SMART* and *SAINT* Bruker AXS Inc., Madison, Wisconsin, USA.

[bb3] Fang, J. M., Selvi, S., Liao, J. H., Slanina, Z., Chen, C. T. & Chou, P. T. (2004). *J. Am. Chem. Soc.* **126**, 3559–3566. 10.1021/ja039237w 15025485

[bb4] Fu, W.-F., Jia, L.-F., Mu, W.-H., Gan, X., Zhang, J.-B., Liu, P.-H. & Cao, Q.-Y. (2010). *Inorg. Chem.* **49**, 4524–4533. 10.1021/ic100094y 20408579

[bb5] Fu, W.-F., Li, H.-F. J., Wang, D.-H., Zhou, L.-J., Li, L., Gan, X., Xu, Q.-Q. & Song, H.-B. (2009). *Chem. Commun.* pp. 5524–5526. 10.1039/b906910k 19753344

[bb6] Goswami, S., Dey, S., Gallagher, J. F., Lough, A. J., García-Granda, S., Torre-Fernández, L., Alkorta, I. & Elguero, J. (2007). *J. Mol. Struct.* **846**, 97–107.

[bb7] Nakatani, K., Horie, S. & Saito, I. (2003). *J. Am. Chem. Soc.* **125**, 8972–8973. 10.1021/ja0350740 15369327

[bb8] Newcome, G. H., Garbis, S. J., Majestic, V. K., Fronczek, F. R. & Chiari, G. (1981). *J. Org. Chem.* **46**, 833–839.

[bb9] Oskui, B. & Sheldrick, W. S. (1999). *Eur. J. Inorg. Chem.* pp. 1325–1333.

[bb10] Sheldrick, G. M. (1996). *SADABS* University of Göttingen, Germany.

[bb11] Sheldrick, G. M. (2008). *Acta Cryst.* A**64**, 112–122. 10.1107/S0108767307043930 18156677

[bb12] Sinha, A., Wahidur Rahaman, S. M., Sarkar, M., Saha, B., Daw, P. & Bera, J. K. (2009). *Inorg. Chem.* **48**, 11114–11122. 10.1021/ic901502n 19877708

